# Alignment of Homologous Chromosomes and Effective Repair of Programmed DNA Double-Strand Breaks during Mouse Meiosis Require the Minichromosome Maintenance Domain Containing 2 (MCMDC2) Protein

**DOI:** 10.1371/journal.pgen.1006393

**Published:** 2016-10-19

**Authors:** Friederike Finsterbusch, Ramya Ravindranathan, Ihsan Dereli, Marcello Stanzione, Daniel Tränkner, Attila Tóth

**Affiliations:** Institute of Physiological Chemistry, Medical Faculty of TU Dresden, Dresden, Germany; University of Sussex, UNITED KINGDOM

## Abstract

Orderly chromosome segregation during the first meiotic division requires meiotic recombination to form crossovers between homologous chromosomes (homologues). Members of the minichromosome maintenance (MCM) helicase family have been implicated in meiotic recombination. In addition, they have roles in initiation of DNA replication, DNA mismatch repair and mitotic DNA double-strand break repair. Here, we addressed the function of MCMDC2, an atypical yet conserved MCM protein, whose function in vertebrates has not been reported. While we did not find an important role for MCMDC2 in mitotically dividing cells, our work revealed that MCMDC2 is essential for fertility in both sexes due to a crucial function in meiotic recombination. Meiotic recombination begins with the introduction of DNA double-strand breaks into the genome. DNA ends at break sites are resected. The resultant 3-prime single-stranded DNA overhangs recruit RAD51 and DMC1 recombinases that promote the invasion of homologous duplex DNAs by the resected DNA ends. Multiple strand invasions on each chromosome promote the alignment of homologous chromosomes, which is a prerequisite for inter-homologue crossover formation during meiosis. We found that although DNA ends at break sites were evidently resected, and they recruited RAD51 and DMC1 recombinases, these recombinases were ineffective in promoting alignment of homologous chromosomes in the absence of MCMDC2. Consequently, RAD51 and DMC1 foci, which are thought to mark early recombination intermediates, were abnormally persistent in *Mcmdc2*^*-/-*^ meiocytes. Importantly, the strand invasion stabilizing MSH4 protein, which marks more advanced recombination intermediates, did not efficiently form foci in *Mcmdc2*^*-/-*^ meiocytes. Thus, our work suggests that MCMDC2 plays an important role in either the formation, or the stabilization, of DNA strand invasion events that promote homologue alignment and provide the basis for inter-homologue crossover formation during meiotic recombination.

## Author Summary

Each chromosome is present in two distinct but homologous copies in diploid organisms. To generate haploid gametes suitable for fertilization, these homologous chromosomes must segregate during meiosis. To ensure correct chromosome segregation, homologous chromosomes must align and become connected by inter-homologue crossovers during early meiosis in most taxa including mammals. Defects in these processes result in infertility and aneuploidies in gametes. Alignment of homologous chromosomes and crossover formation entail generation of DNA double-strand breaks and repair of DNA breaks by meiotic recombination. As part of the repair process, single-stranded DNA ends resulting from DNA breaks invade homologous DNA sequences and use them as repair templates. DNA strand invasion events lead to the alignment of homologous chromosomes, and serve as precursors for crossovers. We discovered that meiotic recombination critically depends on the helicase-related minichromosome maintenance domain containing 2 protein (MCMDC2). MCMDC2 likely promotes the formation and/or stabilization of DNA strand invasion events that connect homologous chromosomes. Thus, MCMDC2 is required for DNA breaks to effectively promote alignment of homologous chromosomes. This work reveals a crucial role for MCMDC2 in recombination in mammals, and constitutes an important step in understanding how recombination establishes connections between homologous chromosomes during meiosis.

## Introduction

Chromosome segregation during the first meiotic division uniquely differs from chromosome segregation during mitosis and the second meiotic division [1,2]. Centromeres belonging to sister chromatids are pulled toward opposite spindle poles during mitosis and the second meiotic division. In contrast, centromeres belonging to homologous chromosomes (homologues) that originate from different parents are pulled to opposite spindle poles during the first meiotic division. This bi-orientation of homologue centromeres requires homologues to pair and become physically linked before segregation [1,2]. In most organisms including mammals, inter-homologue physical linkages are provided by the collaborative action of sister chromatid cohesion and inter-homologue crossovers, the latter of which are formed by meiotic recombination during the first meiotic prophase. Meiotic recombination initiates with the programmed generation of large numbers of DNA double-strand breaks (DSBs) (200–400 per cell in mice and humans) by the SPO11 enzyme [3–7]. This results in SPO11-bound DNA ends at break sites [3,4], which are processed to remove SPO11 from DNA –ends and to produce single-stranded 3´ DNA overhangs [8]. These single-stranded DNA ends attract RecA-like recombinases DMC1 and RAD51, which form “recombinosome” complexes that promote invasion of single-stranded DNA ends into homologous DNA sequences to produce so called displacement-loops (D-loops) [9–11]. It is thought that stable strand invasions preferentially occur into homologues as opposed to sister chromatids during meiosis [12–14]. This inter-homologue bias in the formation of recombination intermediates is thought to ensure that DSBs efficiently promote the recognition and the pairing of homologues based on sequence similarity.

DNA breaks are formed and become repaired within the context of chromosome axes, which are linear proteinaceous chromatin structures that form along cores of chromosomes during meiosis [15–18]. Upon successful homologue pairing, axes of homologues closely align and get incorporated into a meiosis-specific chromatin structure, called the synaptonemal complex. The synaptonemal complex consists of two parallel axes and transverse filaments that connect the axes to a shared central linear protein structure, called the central element [19]. The synaptonemal complex is thought to signal the end of the homologue pairing process [20–22] and promote the repair of DSBs by recombination [16,23–28]. This repair involves DNA synthesis that starts from the 3´end of the invading strands, and uses the invaded homologous sequence as a template [11]. Meiotic recombination-mediated DSB repair has two main pathways with distinct outcomes: reciprocal recombination/crossovers and non-reciprocal recombination/non-crossovers [5,11]. At least one strand invasion on each chromosome is thought to be specially stabilized and turned into a crossover. In contrast, most of the strand invasions are repaired as non-crossovers, which often manifest as gene conversions after the completion of repair.

Correct homologue pairing and crossover formation require finely balanced activities that either stabilize or destabilize strand invasions and resultant recombination intermediates. The BLM helicase has been suggested to destabilize strand invasion intermediates, and this function might be important for error correction of strand invasions and the dissolution of difficult-to-repair recombination intermediates [29–34]. The strand invasion intermediate destabilizing activity of BLM is counteracted by the MutSɣ complex [31,35], which consists of a heterodimer of MSH4 and MSH5 that form clamps around DNA strand invasion intermediates thereby stabilizing them [36]. Accordingly, MSH4 and MSH5 proteins are necessary for the alignment of homologues, homologous synaptonemal complex formation and the efficient completion of DNA repair during meiosis in mammals [37–39]. Putative helicases of the minichromosome maintenance (MCM) protein family have also been implicated in promoting recombination, although MCM proteins were initially discovered as hexameric helicases that are required for the initiation of DNA replication (reviewed in [40]). In particular, three MCM-related *Drosophila* proteins, REC, MEI-217 and MEI-218, form a complex and promote meiotic crossover formation by stabilizing strand invasion intermediates, opposing BLM function and inhibiting the non-homologous end joining repair pathway of DNA break repair [41–43]. Although these proteins are not homologous to MSH4 or MSH5, it was proposed that REC, MEI-217 and MEI-218 substitute for the MutSɣ complex, which is missing from *Drosophila* [43]. MCM8, an orthologue of REC, plays important roles in homologous recombination in plants and vertebrates, where the MutSɣ complex is present [44–48]. However, unlike *Drosophila* REC, vertebrate MCM8 is also important for mitotic recombination and DSB repair [45–48]. Mammalian MCM8 forms a complex with MCM9 in mitotic cells [45,48] and is important for resection of DNA ends at break sites at the initial stages of homologous recombination in mitotic cells [47]. In contrast to MCM8, MCM9 does not play an essential role in meiotic recombination [45]. Curiously, MCM8 is apparently not needed for resection of DNA ends at break sites in meiosis, yet it is important for an as yet undefined recombination step that is essential for efficient homologue alignment and synaptonemal complex formation [45]. This suggests that mammalian MCM8 performs MCM9-independent functions in meiosis, and that like *Drosophila* REC [43], mammalian MCM8 might also have a function in stabilizing DNA strand invasion intermediates in meiosis. Interestingly, the REC interacting MEI-217 and MEI-218 proteins of *Drosophila* also have a predicted orthologue in mammals, called MCMDC2 [43]. Yet, it has not been reported if mammalian MCMDC2 is involved in meiotic recombination. Here we describe the functional analysis of *Mcmdc2*^*-/-*^ mice and show that mouse MCMDC2 is crucial for meiotic recombination and DSB repair. More specifically, we hypothesize that MCMDC2 promotes the formation and/or the stabilization of strand invasion intermediates that permit alignment of homologues.

## Results

### Mcmdc2 is preferentially expressed in the gonads and required for fertility in both sexes

**Fig 1:**
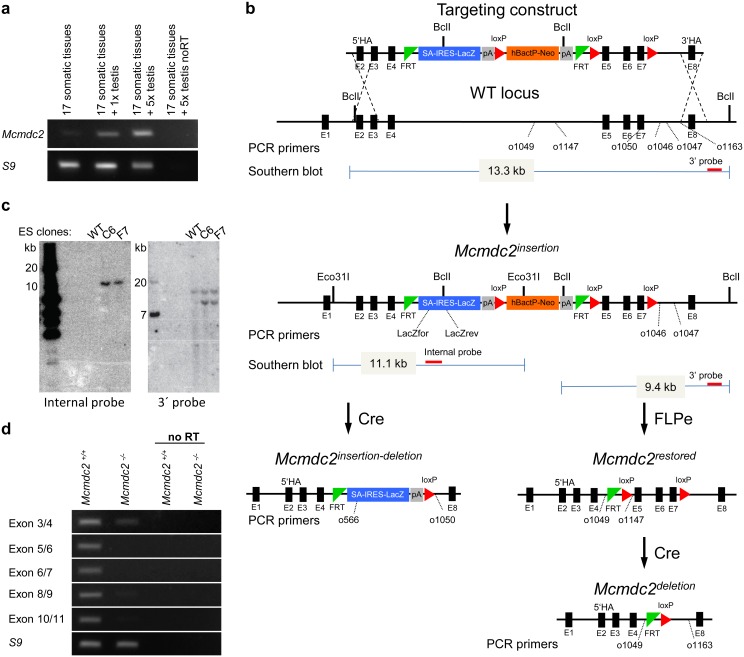
Preferential expression of *Mcmdc2* in the gonads, and *Mcmdc2* targeting in mice. (**a**) Expression of *Mcmdc2* and a “house-keeping” gene (*S9*) in testis and a somatic tissue mix measured by RT-PCR. cDNAs were prepared from four RNA mixtures: (1) Equal amounts of RNAs from 17 somatic tissues (see Materials and Methods for the tissue list) were mixed and 1μg of the resulting mixture was used for RT (17 somatic tissues). (2) Mixture “1” supplemented with testis RNA at a concentration equal to that of the individual somatic RNAs (17 somatic tissues + 1x testis). (3) Mixture “1” supplemented with testis RNA at a concentration equal to five times that of the individual somatic RNAs (17 somatic tissues + 5 x testis) (4) Mixture “3” with no RT (17 somatic tissues + 5xtestis noRT). *Mcmdc2*-specific PCR-products were amplified preferentially from templates that contained testis cDNA. (**b**) *Mcmdc2* targeting strategy. Schematics of the targeting construct, the wild-type (WT) and the modified *Mcmdc2* genomic locus. Black boxes represent exons (not to scale). Recombination at the homology arms (HA) of the targeting construct modifies intron 4 by introducing: 1) an additional exon (SA-IRES-LacZ) that contains a strong splice acceptor site (SA) and poly-adenylation site (left grey box), 2) a transcriptional unit that contains the strong housekeeping human ß-Actin promotor *(hBactP)* driving the neomycin (Neo) resistance gene as a selection marker. This modification of intron 4 also disrupts the *Mcmdc2* open reading frame after the 95th codon *(Mcmdc2*^*insertion*^ allele). Recombination catalyzed by FLPe at FRT sites removes the SA-IRES-LacZ exon and the hBactP-Neo gene, and restores the MCMDC2 ORF (*Mcmdc2*^*restored*^). *Mcmdc2*^*restored*^ is a functional allele that can be disrupted by Cre-mediated recombination between loxP sites (*Mcmdc2*^*deletion*^). Excision of exon 5–7 causes a frameshift after the 80th codon. Cre-mediated recombination between loxP sites of a *Mcmdc2*^*insertion*^ allele results in *Mcmdc2*^*insertion-deletion*^ allele. The positions of PCR-genotyping primers are indicated. Red bars mark the 3`and the internal Southern blot probes; the predicted length of restriction fragments is indicated. (**c**) Southern blot of DNA from wild-type (+/+) and targeted *Mcmdc2*^*+/insertion*^ (*+/i*) embryonic stem cell clones (C6 and F7) that were used to derive two independent mouse lines. DNA was digested with Eco31I and hybridized with an internal probe for LacZ (left panel), or DNA was digested with BclI and hybridized with a 3’ probe (right panel). The blots indicate a single integration of the targeting cassette in the *Mcmdc2* locus. (**d**) RT-PCR was used to detect *Mcmdc2* and "house-keeping" *Rps9 (S9)* transcripts in testes of wild-type and *Mcmdc2*^*-/-*^ (insertion-deletion) mice. Oligo-pairs specific to *Mcmdc2* exon 3 and 4, 5 and 6, 6 and 7, 8 and 9, or 10 and 11 were used.

To address if MCMDC2 could play a role in meiotic recombination we asked if *Mcmdc2* transcripts are present in testis. Thus, we used RT-PCR to assess expression levels of *Mcmdc2* in 17 somatic tissues and testes of mice (Fig 1a). The RT-PCR analysis indicated that *Mcmdc2* transcripts were indeed enriched in testis as compared to somatic tissues (Fig 1a). Furthermore, analysis of public databases (http://www.germonline.org/Homo_sapiens/geneview?gene=ENSG00000178460) [49] showed that human *Mcmdc2* was preferentially expressed in the testis, particularly in spermatocytes. Thus, its expression suggested a role for *Mcmdc2* in meiosis. Given that the *Drosophila* homologs of MCMDC2 are required for crossover formation [50–52], we speculated that mammalian MCMDC2 may also function in meiotic recombination. To test this hypothesis, we attempted to generate antibodies against distinct fragments of mouse MCMDC2 both in rabbit and guinea pig, however none of our antibodies reliably detected MCMDC2, which precluded localization studies of MCMDC2. To directly test the biological functions of MCMDC2 we generated mice where *Mcmdc2* was disrupted after the 4^th^ exon (Fig 1b–1d). The targeting strategy was designed to terminate the 681-amino acid-long MCMDC2 protein after the 95th amino acid (Fig 1b). This was due to the combined effects of the removal of the 5-7^th^ exons (encodes 96–237 amino acids of MCMDC2) causing a frameshift, and the insertion of a strong ectopic splice acceptor site and a transcriptional terminator into the 4^th^ intron. RT- PCR analysis confirmed strongly reduced expression of *Mcmdc2* exons beyond the 4^th^ exon (including exon 8–11, which are not deleted from the genome) in testes of the *Mcmdc2*^*-/-*^ mice (Fig 1d). Even transcripts of exon 3–4, which are upstream of the deletion, were detected at a lower level in *Mcmdc2*^*-/-*^ testes than in wild-type testes. MCMDC2 protein fragments that may be produced from these residual transcripts are unlikely to be functional. This is because deletion of the 5-7^th^ exons would allow only a short 95 amino acid N-terminal fragment to be produced from exons 1–4. Even if alternative splicing generated rare transcripts where sequences from the 4^th^ exon were linked to sequences downstream of the deleted 5-7^th^ exons, protein products of these transcripts would lack most parts of MCMDC2, including the entire conserved MCM-like region (SMART: SM00350, amino acids 177–623 of MCMDC2), because these transcripts would be subject to a frameshift mutation.

**Fig 2:**
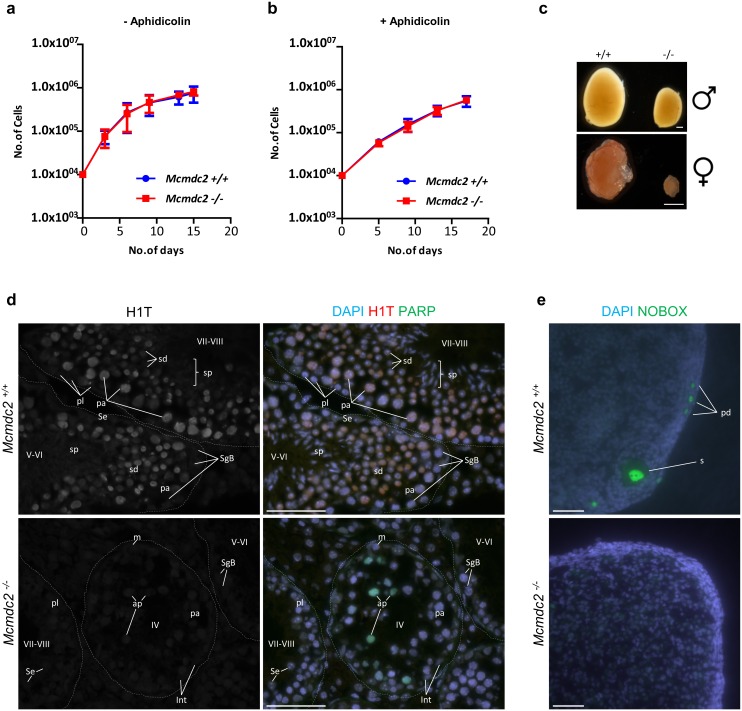
*Mcmdc2*^*-/-*^ mice are deficient in germ cells from late meiotic prophase onwards in both sexes. (**a, b**) Growth curves of five (**a**) or three (**b**) independent lines of *Mcmdc2*^*+/*+^ (+/+) and *Mcmdc2*^*-/-*^ mouse embryonic fibroblasts. Cells were grown either without aphidicolin treatment (**a**) or with aphidicolin treatment for the first 24 hours (**b**), where 1μM aphidicolin was added at day 0. (**a, b**) Cell numbers were determined at the indicated time points in three technical replicates of each fibroblast line. Means and standard deviations of the medians of technical triplicates are shown. Growth curves of *Mcmdc2*^*+/*+^ and *Mcmdc2*^*-/-*^ mouse embryonic fibroblasts are not significantly different (**a**: p = 0.8201, **b**: p = 0.9932, two-way ANOVA test). (**c**) Images of *Mcmdc2*^*+/*+^ (+/+) and *Mcmdc2*^*-/-*^ (-/-) testes (upper panel) and ovaries (lower panel). Scale bars; 500μm. (**d**) Cryosections of testes from adult *Mcmdc2*^*+/+*^ and *Mcmdc2*^*-/-*^ mice. DNA was detected by DAPI, histone H1T (marker of spermatocytes after mid-pachytene) and nuclear cleaved PARP1 (marker of apoptotic cells) were detected by immunostaining. Outlines of testis tubules are marked by dashed lines. The upper panels of **d** show stage V-VI and VII-VIII wild-type testis tubules, which contain several layers of germ cells at distinct spermatogenic stages: Sertoli cells (Se), spermatogonia B (SgB, stage V-VI), preleptotene (pl, stage VII-VIII), mid-pachytene (pa, stage V-VI), late-pachytene (pa, stage VII-VIII) spermatocytes, post-meiotic spermatids (sd) and spermatozoa (sp). Lower panels of **d** show that *Mcmdc2*^*-/-*^ meiocytes underwent apoptosis at a stage corresponding to wild-type mid-pachytene in stage IV tubules. Consequently, spermatocytes were not found in the inner layers of testis tubules beyond stage IV, and post-meiotic spermatids and spermatozoa were also missing from *Mcmdc2*^*-/-*^ testes. To illustrate this, stage IV, V-VI and VII-VIII tubules of *Mcmdc2*^*-/-*^ mice are shown. Apoptotic (ap) and non-apoptotic early-mid pachytene (pa) spermatocytes are shown in the stage IV tubule, which was identified by the presence of mitotic intermediate spermatogonia (m) and intermediate spermatogonia (Int). Stage V-VI and VII-VIII tubules contain somatic Sertoli cells (Se) and spermatogonia B (SgB) or preleptotene (pl) spermatocytes, respectively, but more advanced spermatogenic cells are missing. Due to elimination at mid-pachytene, histone H1T positive cells are missing from *Mcmdc2*^*-/-*^ testis tubules. (**e**) NOBOX (oocyte marker) was detected by immunofluorescence on cryosections of ovaries from 6-week-old mice. DNA was stained by DAPI. Oocytes in primordial (pd) and secondary (s) follicles are shown in the section of a wild-type ovary. In contrast, oocytes are not detected in the shown *Mcmdc2*^*-/-*^ ovary section. (**d, e**) Scale bars; 50μm.

*Mcmdc2*^*-/-*^ mice were viable and did not show any obvious somatic defects. Although the previously published *Mcm8*^*-/-*^ and *Mcm9*^*-/-*^ mice were viable, *Mcm8*^*-/-*^ and *Mcm9*^*-/-*^ mouse embryonic fibroblasts (MEFs) displayed slow growth and sensitivity to the DNA replication inhibitor aphidicolin [45]. These phenotypes were attributed to the functions of MCM8 and MCM9 in mitotic homologous recombination. To test if MCMDC2 had a defect in mitotic cell cycle due to a possible function in mitotic recombination we established MEFs from *Mcmdc2*^*-/-*^ and *Mcmdc2*^*+/+*^ litter-mate embryos. MEFs of *Mcmdc2*^*-/-*^ and wild-type mice did not differ significantly in their growth rate or in their sensitivity to aphidicolin (Fig 2a and 2b). This suggests that unlike MCM8 and MCM9, MCMDC2 does not play an important role during mitotic growth.

Importantly, while we observed no obvious somatic defects, both sexes of *Mcmdc2*^*-/-*^ mice were infertile (no pups after 113 breeding weeks, n = 3 males and n = 3 females) because both oogenesis and spermatogenesis were blocked (Fig 2c–2e). Ovaries of 6 weeks old *Mcmdc2*^*-/-*^ females were atrophic, barely discernible, and completely devoid of oocytes (n = 3 mice, Fig 2c and 2e). This was due to an apparent loss of oocytes perinatally or soon after birth, as oocytes were still present in ovaries of fetal and newborn *Mcmdc2*^*-/-*^ mice. Spermatogenesis takes place within testis tubules, which can be found at 12 distinct stages of the seminiferous epithelial cycle (stages I-XII). Each stage is identified by the distinct combinations of spermatogenic cells found within [53]. We observed no late prophase spermatocytes and postmeiotic cells in *Mcmdc2*^*-/-*^ males. This was due to apoptosis of spermatocytes in epithelial cycle stage IV testis tubules (Fig 2d); in the wild type, stage IV tubules contain spermatocytes at the mid pachytene stage. Consistent with a complete elimination of spermatocytes at stage IV no histone H1T (a late prophase marker [54]) positive cells were found in *Mcmdc2*^*-/-*^ testis tubules (n>200 tubules, Fig 2d). In contrast, histone H1T was increasingly expressed in wild-type spermatocytes beyond stage IV as expected.

### Mcmdc2 is required for efficient synaptonemal complex formation

**Fig 3:**
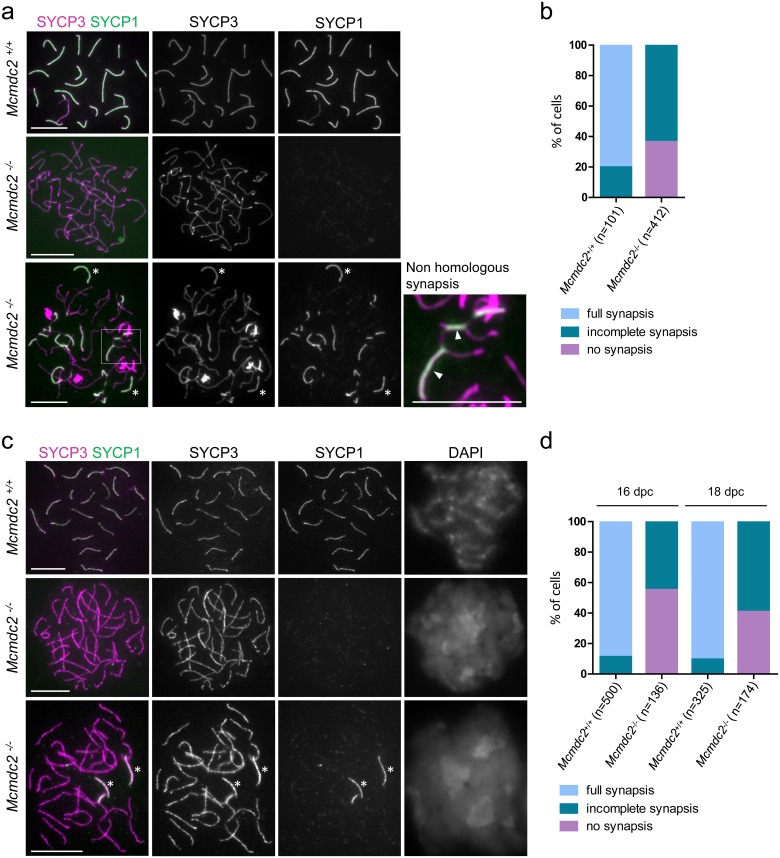
Synaptonemal complex formation is defective in *Mcmdc2*^*-/-*^ mice. (**a, c**) SYCP3 (axis marker) and SYCP1 (synaptonemal complex marker) were detected by immunofluorescence on nuclear surface spreads of *Mcmdc2*^*+/+*^ pachytene and *Mcmdc2*^*-/-*^ zygotene-pachytene spermatocytes (**a**) and oocytes (**c**). Two distinct categories of *Mcmdc2*^*-/-*^ zygotene-pachytene meiocytes were found. They either had no synapsis with no or weak punctate SYCP1 along unsynapsed axes (middle rows, **a** and **c***)*, or stretches of SYCP1 formed between some chromosomes that have managed to synapse (bottom panels, **a** and **c**). The spermatocyte shown in the bottom panels of **a** illustrates the maximum extent of synaptonemal complex formation observed in *Mcmdc2*^*-/-*^ spermatocytes. Although some chromosomes evidently managed to fully synapse (asterisks in **a** and **c**) most synaptonemal complexes are incomplete and often form between apparently non-homologous axes (**a** inset, arrowheads mark synaptonemal complex stretches between apparently non-homologous chromosomes that have different lengths). Note that X and Y sex chromosomes are synapsed only in their short PAR regions in spermatocytes (**a**, top). Scale bars; 10μm. (**b, d**) Quantification of synaptonemal complex formation in *Mcmdc2*^*+/+*^ and *Mcmdc2*^*-/-*^ spermatocytes (**b**) or oocytes (**d**). Spermatocytes were collected from testes of adult mice, and oocytes were collected from the ovaries of 16 or 18 days post coitum (dpc) fetuses. Synaptonemal complex development was assessed by detecting SYCP3 (axis marker) and SYCP1 (synaptonemal complex marker) on nuclear spreads of meiocytes from littermate pairs of wild-type and *Mcmdc2*^*-/-*^ mice. Three categories of synaptonemal complex formation were distinguished in cells with fully formed continuous axes: 1) No synapsis (characterized by either no SYCP1 stain or punctate weak SYCP1 stain along unsynapsed axes), 2) incomplete synapses (stretches of axes have formed but there is at least one chromosome without fully completed synaptonemal complex) or 3) full synapsis (all chromosomes are fully synapsed except the male sex chromosomes). Counted numbers of cells are indicated (n).

Elimination of meiocytes at the observed stages indicated a possible defect in meiotic recombination in *Mcmdc2*^*-/-*^ mice. Specifically, persistent asynapsis and failure in DNA break repair is known to elicit elimination of spermatocytes and oocytes in stage IV testis tubules and perinatal ovaries, respectively [55–58]. Therefore we examined synaptonemal complex formation and markers of meiotic recombination in *Mcmdc2*^*-/-*^ meiocytes. While chromosome axes readily formed (as judged by SYCP3 staining), synaptonemal complex formation was severely defective in *Mcmdc2*^*-/-*^ meiocytes (as shown by disrupted SYCP1 localization along chromosome axes) (Fig 3). In the most advanced stages full axes formed along the core of each chromosome in *Mcmdc2*^*-/-*^ spermatocytes. Fully assembled chromosome axes can be observed from late zygotene to diplotene in wild-type spermatocytes. Given that *Mcmdc2* ^*-/-*^ spermatocytes were eliminated at a stage equivalent to wild-type mid-pachytene we postulate that *Mcmdc2*^*-/-*^ spermatocytes with fully formed axes reached a prophase stage equivalent to wild-type late zygotene to mid pachytene, hence we refer to this stage in *Mcmdc2* ^*-/-*^ spermatocytes as late zygotene-pachytene.

In the wild type, all late zygotene spermatocytes had partially synapsed chromosomes and by early pachytene all autosomes fully synapsed (n = 101 cells, of which 20% were late zygotene and 80% were early pachytene, Fig 3b). In contrast, in *Mcmdc2* ^*-/-*^ spermatocytes, synapsis of all chromosomes was never observed (n>500 spermatocytes) as chromosome axes were unaligned and remained mostly unsynapsed in late zygotene-pachytene. The transverse filament component SYCP1, which marks synapsed axes [59], was detectable in a punctate pattern at very low levels along unsynapsed chromosome axes, or was detected only at a few foci at intersections of chromosome axes in 36% of late zygotene-pachytene *Mcmdc2* ^*-/-*^ spermatocyte nuclear spreads (n = 412 cells, Fig 3b). In the rest of the spermatocytes, stretches of SYCP1 were detected along sections of juxtaposed axes, but the numbers of these synaptonemal complexes were generally low (a median number of 4 SYCP1 stretches in each spermatocyte, n = 131 cells). Chromosomes with different axis lengths were engaged in synapsis with each other, and chromosomes formed synapsis with more than one partner indicating that synaptonemal complexes frequently formed between non-homologous chromosomes (53% of synaptonemal complex stretches were unequivocally identified as non-homologous, n = 232 synaptonemal complex stretches in 62 spermatocytes). Nevertheless, in a significant fraction of cells (14 out of 116 cells) we observed apparently fully synapsed chromosomes (median 1 fully synapsed chromosome, range 1–4). We observed a similar chromosome alignment and synaptonemal complex formation defect in *Mcmdc2*^*-/-*^ oocytes that were collected from 16 or 18dpc fetuses. At these stages most wild-type oocytes were in early (16dpc) or late (18dpc) pachytene stages, and all chromosomes in oocytes with fully formed axes were either fully synapsed (16dpc: 89%, n = 500, 18dpc: 90%, n = 325 oocytes) or partially synapsed (16dpc: 11%, 18dpc: 10%) (Fig 3d). In contrast, a large fraction of *Mcmdc2* ^*-/-*^ oocytes with fully formed axes (16dpc: 55%, n = 136, 18dpc: 41%, n = 174 oocytes) lacked synapsis completely or formed only punctate/very short stretches of synaptonemal complexes at intersections of axes (Fig 3d). Stretches of synaptonemal complexes formed in 45% (16dpc) or 59% (18dpc) of oocytes. Among those oocytes with SYCP1 stretches, the median number of stretches was 4 at 16dpc (n = 61) and 5 at 18dpc (n = 103). We also observed apparently fully synapsed chromosomes in 10 out of 61 (16dpc) or 73 out of 103 (18dpc) oocytes where SYCP1 stretches were observed. In cells which had fully synapsed chromosomes, a median number of 1 fully synapsed chromosome was observed at 16dpc (n = 10 oocytes) and 2 at 18dpc (n = 73). The highest number of fully synapsed chromosomes we observed was 8. We detected low levels of punctate SYCP1 signals along unsynapsed axes in *Mcmdc2*^*-/-*^ meiocytes in both sexes (male: Fig 3a, female: Fig 3c), although this SYCP1 staining pattern was more obvious in oocytes. This suggested that synaptonemal complex transverse filament assembly was initiated, although synaptonemal complex formation mostly failed in the absence of effective homologue alignment in *Mcmdc2* ^*-/-*^ meiocytes. A similar weak association of SYCP1 with unsynapsed chromosome axes has been described in DNA strand invasion-defective *Dmc1*^*-/-*^ and *Hop2*^*-/-*^ meiocytes [60]. Thus, SYCP1 accumulation along unsynapsed chromosome axes may be a general phenomenon that can occur when synaptonemal complex formation is initiated but cannot be completed along unpaired chromosome axes. Taken together, these observations showed that MCMDC2 is critical for homologue alignment and synaptonemal complex formation in both sexes.

### MCMDC2 is required for the repair of programmed meiotic DNA breaks

**Fig 4:**
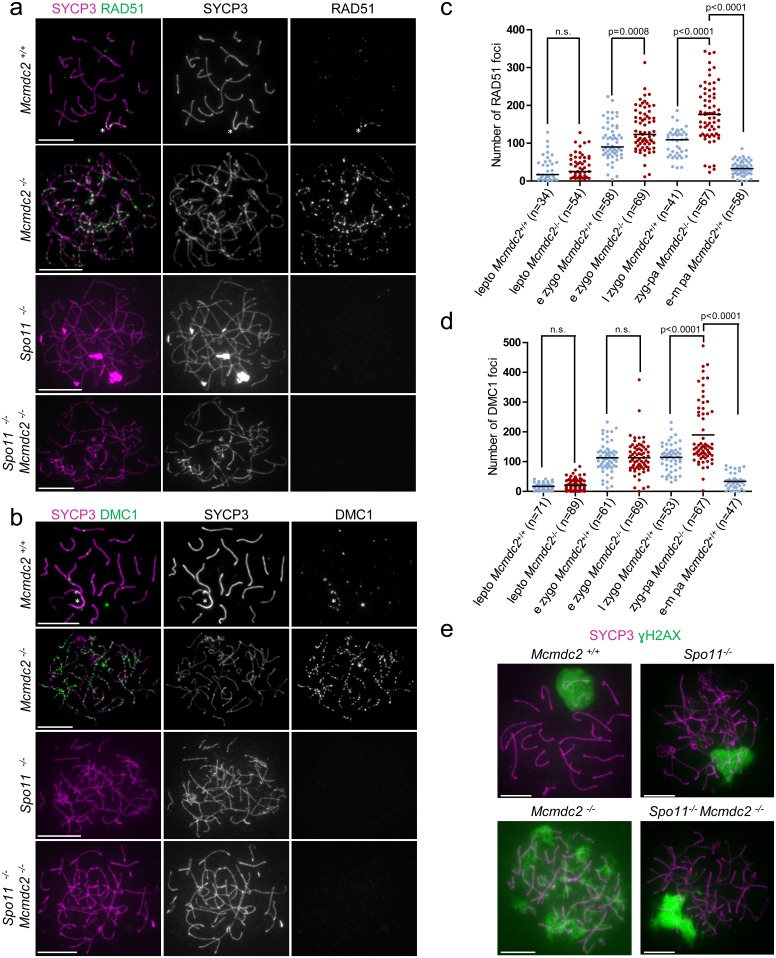
RAD51 and DMC1 foci persist in *Mcmdc2*^*-/-*^ *s*permatocytes. (**a, b, e**) Immunostaining showing SYCP3 together with RAD51 (**a**), DMC1 (**b**) or γH2AX (**e**) on nuclear surface spreads of pachytene *Mcmdc2*^*+/+*^, late zygotene-pachytene *Mcmdc2*^*-/-*^, *Spo11*^*-/-*^, and *Spo11*^*-/-*^ *Mcmdc2*^*-/—*^spermatocytes. RAD51 and DMC1 foci are present at comparatively high density along the axes of unsynapsed sex chromosomes (**a, b**, asterisk), and are largely absent from synapsed autosomes of *Mcmdc2*^*+/+*^ spermatocytes. Both RAD51 and DMC1 foci are present in high numbers along the unpaired axes of *Mcmdc2*^*-/-*^ spermatocytes. Absence of RAD51 and DMC1 foci is shown in *Spo11*^*-/-*^ and *Spo11*^*-/-*^ *Mcmdc2*^*-/—*^spermatocytes. (**e**) γH2AX preferentially accumulates on the partially synapsed sex chromosomes of the *Mcmdc2*^*+/+*^ spermatocyte. γH2AX associates with chromatin throughout the nucleus in the *Mcmdc2*^*-/-*^ spermatocytes. γH2AX is largely restricted to the sex chromatin in wild-type pachytene spermatocytes, and to pseudo-sex bodies in *Spo11*^*-/-*^ and *Spo11*^*-/-*^ *Mcmdc2*^*-/—*^spermatocytes. Scale bars; 10μm. (**c, d**) Numbers of RAD51 (**c**) or DMC1 (**d**) foci are shown in leptotene (lepto), early zygotene (e zygo) in *Mcmdc2*^*+/+*^ and *Mcmdc2*^*-/-*^, late zygotene (l zygo) and early-mid pachytene (e-m pa) in *Mcmdc2*^*+/+*^ and zygotene-pachytene (zyg-pa) in *Mcmdc2*^*-/-*^ spermatocytes. Median numbers of foci are marked, and n corresponds to the number of analyzed spermatocytes in three pooled experiments. DMC1 and RAD51 foci numbers are significantly higher in zygotene-pachytene *Mcmdc2*^*-/-*^ spermatocytes than in late-zygotene or early-mid-pachytene *Mcmdc2*^*+/+*^ spermatocytes (Mann Whitney test).

The observed defects in homologue alignment and synaptonemal complex formation suggested that early stages of recombination may be defective in the absence of MCMDC2. Single-stranded DNA ends that are produced after DSB formation are bound by recombinases RAD51 and DMC1, which form foci along chromosome axes. These foci have been defined as early recombination nodules by electron-microscopy and are thought to represent recombinosome complexes [61–66]. DMC1 and RAD51 promote strand-invasion of DNA ends into homologues [9–11]. This leads to synaptonemal complex formation, and as DNA repair progresses, the early recombinosomes/recombination nodules lose DMC1 and RAD51 and progress to become transitional recombinosomes/nodules [61,62,64,65]. Hence, quantification of DMC1 and RAD51 foci is informative about the number of unrepaired DNA breaks involved in early stages of recombination in meiocytes. We found that foci of both RAD51 and DMC1 accumulate with similar kinetics in wild-type and *Mcmdc2*^*-/-*^ spermatocytes in leptotene and early zygotene stages of prophase (Fig 4c and 4d). Numbers of RAD51 and DMC1 foci dropped as wild-type spermatocytes progressed to early-mid pachytene. In contrast, high numbers of RAD51 and DMC1 foci persisted in late zygotene-pachytene *Mcmdc2*^*-/-*^ spermatocytes. The high RAD51 and DMC1 foci numbers in *Mcmdc2*^*-/-*^ spermatocytes required SPO11 (Fig 4a and 4b). This observation is consistent with the idea that a delay in the repair of SPO11-generated programmed DSB breaks causes accumulation of RAD51 and DMC1 foci in *Mcmdc2*^*-/-*^ spermatocytes. To further test if meiotic DSB repair is delayed in the absence of MCMDC2 we detected phospho-serine 139 histone H2AX (ɣH2AX), which accumulates on chromatin in response to unrepaired DNA breaks and asynapsis in meiotic cells. ɣH2AX decorated chromatin in leptotene and zygotene stages but largely disappeared from autosomal chromatin due to progression of DSB repair and synapsis in wild-type cells. It remained associated only with the chromatin of the largely unsynapsed sex chromosomes, which form the sex body at the early pachytene stage (Fig 4e). In stark contrast to wild-type spermatocytes, late zygotene-pachytene *Mcmdc2*^*-/-*^ spermatocytes failed to form sex bodies and ɣH2AX persisted on autosomal chromatin (n>200 cells). Persistent widespread ɣH2AX accumulation on chromatin was dependent on SPO11, as *Mcmdc2*^*-/-*^ *Spo11*^*-/-*^ spermatocytes formed only more localized ɣH2AX-rich chromatin domains, so called pseudo-sex bodies, which are characteristic of mutants defective in programmed DSB formation (Fig 4e) [55,67].

**Fig 5:**
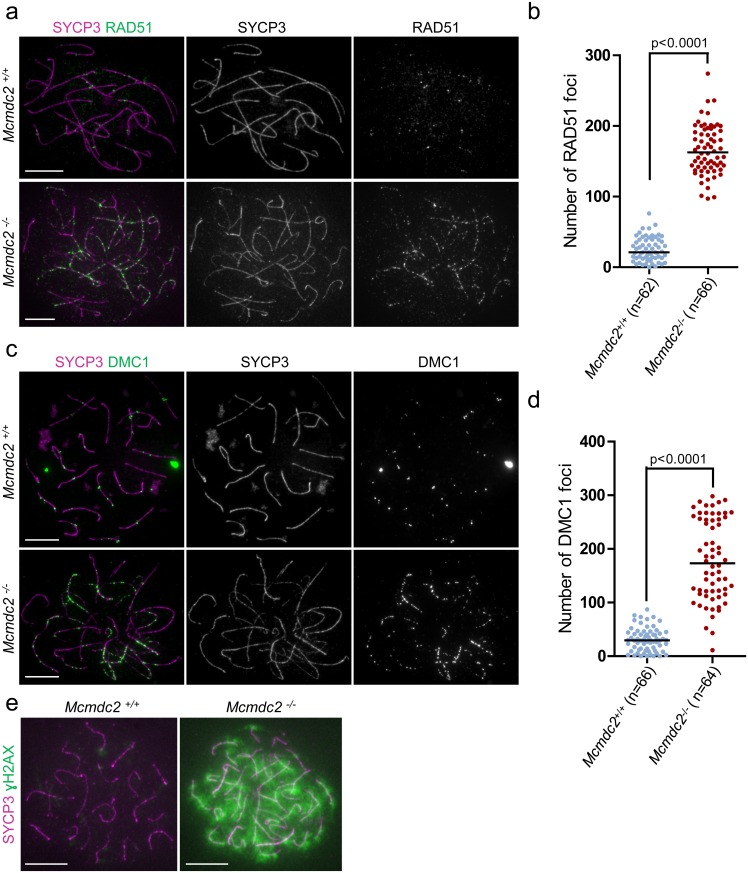
RAD51 and DMC1 foci persist in *Mcmdc2*^*-/-*^ oocytes. (**a, c, e)** Immunostaining of SYCP3 along with RAD51 (**a**), DMC1 (**c**) or γH2AX (**e**) on nuclear surface spreads of pachytene *Mcmdc2*^*+/+*^, or zygotene-pachytene *Mcmdc2*^*-/-*^ oocytes. Oocytes were collected from the ovaries of littermate fetuses at 18dpc, which is a time point when most wild-type oocytes are in the late pachytene stage. RAD51 and DMC1 foci are largely absent from synapsed chromosomes in *Mcmdc2*^*+/+*^ oocytes. Both RAD51 and DMC1 foci are present in high numbers along the unpaired axes of *Mcmdc2*^*-/-*^ oocytes. (**e**) γH2AX is largely absent from the synapsed chromosomes of the *Mcmdc2*^*+/+*^ oocyte. γH2AX associates with chromatin throughout the nucleus in the *Mcmdc2*^*-/-*^ oocyte. Scale bars; 10μm. (**b, d**) Numbers of RAD51 (**b**) or DMC1 (**d**) foci are shown in *Mcmdc2*^*+/+*^ and *Mcmdc2*^*-/-*^ oocytes at 18dpc. Median numbers of foci are marked, and n corresponds to the number of analyzed oocytes in two pooled experiments. DMC1 and RAD51 foci numbers are significantly higher in *Mcmdc2*^*-/-*^ than in *Mcmdc2*^*+/+*^ oocytes (Mann Whitney test).

We found a similar delay in meiotic recombination in *Mcmdc2*^*-/-*^ oocytes (Fig 5). We observed low numbers of DMC1 foci (median 29.5 foci, n = 66 cells) or RAD51 foci (median 21 foci, n = 62 cells) in late pachytene wild-type oocytes at 18dpc (Fig 5b and 5d). Correspondingly, the chromatin of pachytene oocytes was depleted of ɣH2AX (n>100 oocytes, Fig 5e). In contrast, both foci of RAD51 (median 162.5 foci, n = 66 cells) and DMC1 (median 173 foci, n = 64 cells) persisted along unsynapsed axes and ɣH2AX accumulated to high levels throughout the genome (n>100 oocytes, Fig 5e) in oocytes from *Mcmdc2*^*-/-*^ females.

The combination of these observations suggests that MCMDC2 is not required for DSB formation or the loading of recombinases on single-stranded DNA ends. Yet, RAD51/DMC1 appears to be ineffective in promoting homologue alignment and DNA break repair is severely impaired in the absence of MCMDC2.

### Formation of MSH4- and MLH1-marked recombination intermediates requires MCMDC2

**Fig 6:**
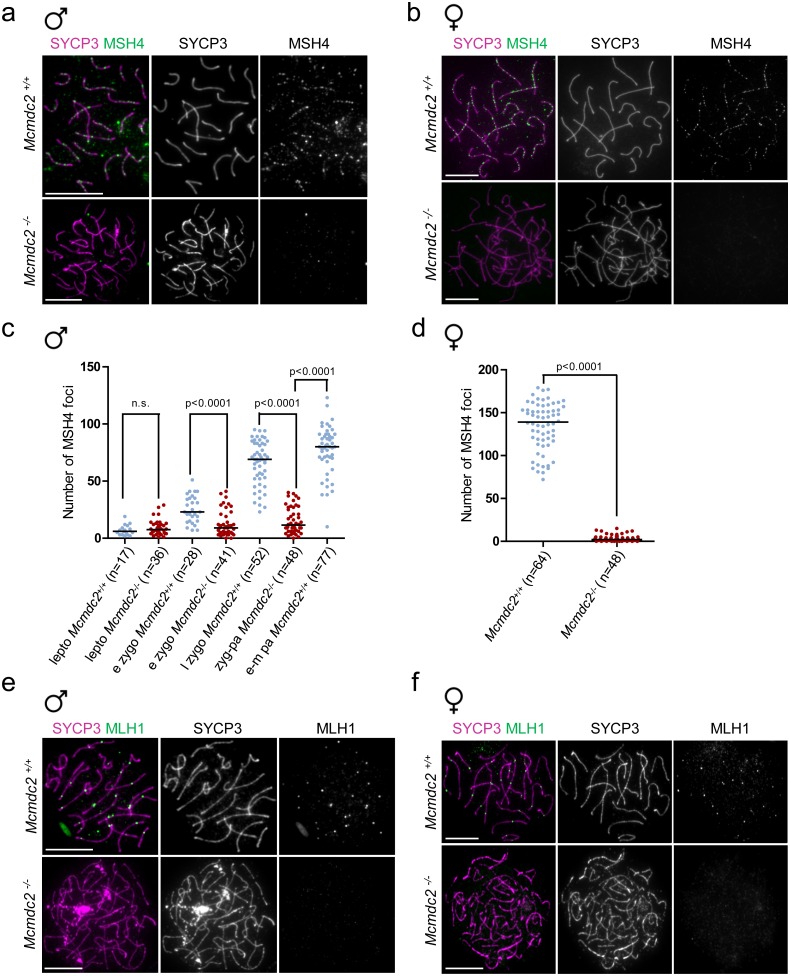
MutSγ and MutLγ foci formation are defective in *Mcmdc2*^*-/-*^ meiocytes. (**a, b, e, f**) Immunostaining of SYCP3 together with MSH4 (**a, b**) or MLH1 (**e, f**) on nuclear surface spreads of pachytene *Mcmdc2*^*+/+*^ or zygotene-pachytene *Mcmdc2*^*-/-*^ meiocytes. (**a, b**) MSH4 foci are readily detected along synapsed axes of pachytene spermatocytes and oocytes (16dpc). MSH4 foci numbers are much lower in *Mcmdc2*^*-/-*^ meiocytes. (**e, f**) Typically, a single MLH1 focus is detected along each synapsed axis pair of *Mcmdc2*^*+/+*^ pachytene spermatocytes and oocytes (from ovaries of newborn mice). MLH1 foci are not present along the unsynapsed axes of *Mcmdc2*^*-/-*^ meiocytes. Scale bars; 10μm. (**c, d**) Numbers of MSH4 foci in *Mcmdc2*^*+/+*^ and *Mcmdc2*^*-/-*^ spermatocytes and oocytes. (**c**) Spermatocytes were examined at leptotene (lepto), early zygotene (e zygo) in *Mcmdc2*^*+/+*^ and *Mcmdc2*^*-/-*^, late zygotene (l zygo) and early-mid pachytene (e-m pa) in *Mcmdc2*^*+/+*^ and zygotene-pachytene (zyg-pa) in *Mcmdc2*^*-/-*^ mice. MSH4 foci numbers are significantly lower in *Mcmdc2*^*-/-*^ than in *Mcmdc2*^*+/+*^ spermatocytes from early-zygotene stage onwards (Mann Whitney test). (**d**) Oocytes with fully formed axes (late zygotene and early pachytene) were examined from fetal ovaries at the 16dpc developmental time point. MSH4 foci numbers are significantly lower in *Mcmdc2*^*-/-*^ than in *Mcmdc2*^*+/+*^ oocytes (Mann Whitney test). (**c, d**) Median numbers of foci are marked, and n corresponds to the number of analyzed meiocytes in two pooled experiments.

In wild-type meiosis, successful homologue alignment is accompanied by the appearance of axis-associated MSH4 foci that are thought to represent MSH4/5 (MutSγ) complexes within transitional recombinosomes/recombination nodules [38,61,64]. MutSγ is necessary for robust homologue pairing and alignment, most likely because it stabilizes strand invasion intermediates [23,36–39]. Hence, MSH4 foci are inferred to mark stabilized post-strand invasion recombination intermediates that are needed for efficient homologue alignment. Therefore, we tested if MSH4 forms axis-associated foci in *Mcmdc2*^*-/-*^ meiocytes (Fig 6a–6d). MSH4 foci numbers were significantly lower in *Mcmdc2*^*-/-*^ spermatocytes (median 11.5 foci, in late zygotene-pachytene cells, n = 48) and oocytes (median two foci in 16dpc late zygotene-pachytene oocytes, n = 48) than in wild-type meiocytes (median 80 foci in early-mid pachytene spermatocytes, n = 49, median 139 foci in 16dpc late zygotene and pachytene oocytes, n = 64).

MSH4 foci numbers were marginally higher in late zygotene-pachytene than leptotene *Mcmdc2*^*-/-*^ spermatocytes (p = 0.0138, Mann Whitney test). This may indicate that MSH4-marked intermediates still form with low efficiency in the absence of MCMDC2. However, we observed an increasing punctate anti-MSH4 signal throughout the nuclei of wild-type meiocytes upon progression to pachytene (see Fig 6a upper panel). This pan-nuclear signal is unlikely to represent MutSγ complexes bound to recombination intermediates. Thus, a “background” anti-MSH4 signal in *Mcmdc2*^*-/-*^ spermatocytes may provide an explanation for the low MSH4 foci counts, which show a small increase upon progression from leptotene to late zygotene-pachytene. Regardless, the strongly reduced MSH4 foci numbers of *Mcmdc2*^*-/-*^ meiocytes suggest severe impairment in MutSγ function and/or in a recombination step that precedes recruitment of MutSγ to recombination intermediates.

Most MSH4-marked intermediates are repaired as non-crossovers in late pachytene, but a minority of them (at least one per homologue pair and on average 23 per cell) is thought to develop into MLH1-marked late recombinosomes (defined as late recombination nodules by electron-microscopy) [61,62,64,65,68], which are sites of future crossovers [5,69,70]. Consistent with an impairment in MutSγ-containing recombinosome formation, and consistent with the elimination of spermatocytes in mid-pachytene, we found no MLH1 foci in *Mcmdc2*^*-/-*^ spermatocytes (n = 35 spermatocytes, Fig 6e). We also detected a similar defect in MLH1 foci formation in *Mcmdc2*^*-/-*^ oocytes that had full axis (n = 23 oocytes at 18dpc, and n = 47 oocytes at 20.5dpc/newborn, Fig 6f). Thus, the recombination defect in *Mcmdc2*^*-/-*^ meiocytes ultimately prevents the formation of MLH1 foci, which likely represent precursors of a large majority of meiotic crossovers.

### Inhibition of non-homologous synapsis formation is dependent on SPO11 in the absence of MCMDC2

**Fig 7:**
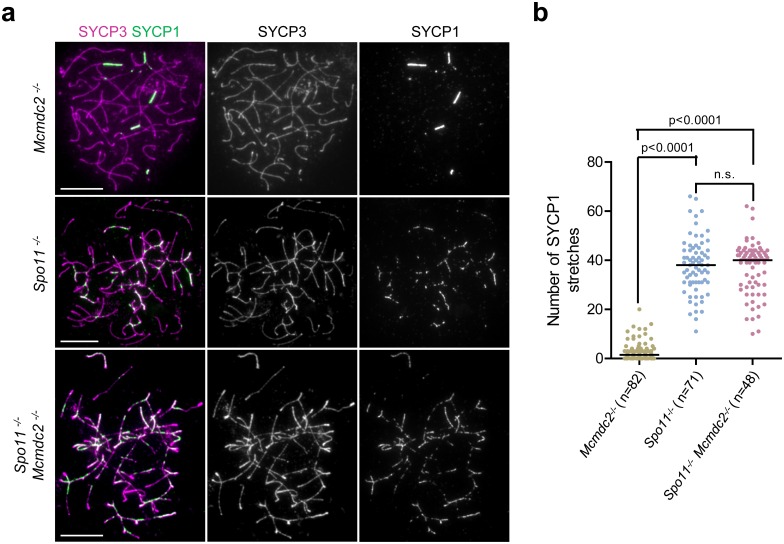
MCMDC2 is not required for extensive non-homologous synaptonemal complex formation in the *Spo11*^*-/-*^ background. (**a**) SYCP3 (axis marker) and SYCP1 (synaptonemal complex marker) were detected by immunofluorescence on nuclear surface spreads of zygotene-pachytene *Mcmdc2*^*-/-*^, *Spo11*^*-/-*^ or *Spo11*^*-/-*^ *Mcmdc2*^*-/—*^spermatocytes. Whereas comparatively few synaptonemal complex stretches are detected in the *Mcmdc2*^*-/-*^spermatocyte, extensive non-homologous synaptonemal complex formation is seen in the *Spo11*^*-/-*^ or *Spo11*^*-/-*^ *Mcmdc2*^*-/—*^spermatocytes. Scale bars; 10μm (**b**) Quantification of SYCP1 stretch numbers in zygotene-pachytene spermatocytes with fully condensed chromosome axes of the indicated genotypes. The numbers of synaptonemal complex stretches is significantly higher in *Spo11*^*-/-*^ or *Spo11*^*-/-*^ *Mcmdc2*^*-/—*^spermatocytes than in *Mcmdc2*^*-/-*^ (Mann Whitney test). The numbers of synaptonemal complex stretches are not significantly different in *Spo11*^*-/-*^ or *Spo11*^*-/-*^ *Mcmdc2*^*-/—*^spermatocytes (p = 0.8639, Mann Whitney test). Median numbers of foci are marked, and n corresponds to the number of analyzed spermatocytes in two (*Spo11*^*-/-*^ or *Spo11*^*-/-*^ *Mcmdc2*^*-/-*^) or three (*Mcmdc2*^*-/-*^) pooled experiments.

While analyzing synaptonemal complexes in *Mcmdc2*^*-/-*^ meiocytes, we noted that synaptonemal complex formation was more severely affected in *Mcmdc2*^*-/-*^ meiocytes than in *Spo11*^*-/-*^ meiocytes (Fig 7). *Spo11*^*-/-*^ meiocytes lack programmed DNA breaks, and thus fail in homologue alignment and homologous synaptonemal complex formation. Nevertheless, synaptonemal complexes extensively formed between non-homologous chromosomes often creating a meshwork of interconnected chromosomes in *Spo11*^*-/-*^ meiocytes of the most advanced stages (Fig 7a middle panel). Our observations suggest that MCMDC2 is needed for meiotic DSB repair and progression beyond the early stages of recombination. Thus, early recombination intermediates may inhibit non-homologous synaptonemal complex formation in *Mcmdc2*^*-/-*^ meiocytes. Alternatively, it is possible that MCMDC2 has a DSB-independent function that is needed for non-homologues synaptonemal complex formation when homologue alignment is defective. To distinguish between these possibilities we tested the epistatic relationship between *Mcmdc2* and *Spo11*. We reasoned that if non-homologous synapsis formation was similarly limited in *Mcmdc2*^*-/-*^ and *Spo11*^*-/-*^ *Mcmdc2*^*-/-*^ meiocytes then this would indicate a DSB-independent role for MCMDC2 in non-homologous synaptonemal complex formation. Conversely, extensive non-homologous synaptonemal complex formation in *Spo11*^*-/-*^ *Mcmdc2*^*-/-*^ meiocytes would indicate a role for SPO11 and SPO11-dependent recombination intermediates in the inhibition of non-homologous synaptonemal complex formation in *Mcmdc2*^*-/-*^ meiocytes. We found that *Mcmdc2*^*-/-*^ did not significantly reduce non-homologous synaptonemal complex formation in a *Spo11*^*-/-*^ background, and accordingly, we observed significantly more non-homologous synaptonemal complex stretches in *Spo11*^*-/-*^ (p = 0.0001, Mann Whitney test) and *Spo11*^*-/-*^ *Mcmdc2*^*-/-*^ (p = 0.0001, Mann Whitney test) spermatocytes than in *Mcmdc2*^*-/-*^ spermatocytes (Fig 7b). Thus, MCMDC2 is not required for non-homologous synapsis that forms in the absence of SPO11 and programmed DSBs. We conclude that *Spo11* is epistatic to *Mcmdc2* in “erroneous” non-homologous synaptonemal complex formation, and that SPO11-dependent recombination intermediates, which fail to promote homologous synaptonemal complex formation in the absence of MCMDC2, most likely interfere with the formation of extensive non-homologous synapsis in *Mcmdc2*^*-/-*^ spermatocytes.

## Discussion

### MCMDC2 is needed for early steps in recombination mediated DNA DSB repair

Our work revealed that the MCM domain-containing protein MCMDC2 is essential for meiotic recombination, and hence gametogenesis. In contrast to MCM8 and MCM9, which have been implicated in homologous recombination during stalled replication fork restart and interstrand crosslink repair in mitotically growing cells [45–48], we found no evidence of an important role for MCMDC2 in mitotic cells (Fig 2a and 2b). We found that *Mcmdc2*^*-/-*^ meiocytes were defective in recombination-mediated repair of programmed meiotic DSBs, alignment of homologues, and synaptonemal complex formation.

DSB repair and synaptonemal complex formation are mutually dependent on each other in mammalian meiosis [24–28,37–39,60,71,72]. Thus, the severe synapsis formation defect observed could either be a cause or a consequence of the failed DNA DSB break repair in *Mcmdc2*^*-/-*^ meiocytes. We favor the hypothesis that the primary role of MCMDC2 is in DSB repair and not in synaptonemal complex formation. In support of this hypothesis, while synaptonemal complex formation is not required for correct alignment of homologue axes [24–28], MCMDC2 and initial steps of recombination that involve the formation of stable inter-homologue strand invasion intermediates are required [37–39,60,71,72]. This implicates MCMDC2 in early recombination steps that are needed for homologue pairing.

Additional support is provided by the observation that DNA DSB repair seems to progress further in synaptonemal complex-defective mutants than in *Mcmdc2*^*-/-*^ meiocytes. RAD51/DMC1-marked early recombinosomes seem to develop into MSH4-marked transitional recombinosomes in mutant spermatocytes that lack structural components of the synaptonemal complex [24–26]. MSH4 is thought to stabilize inter-homologue recombination intermediates [11,23,35,36], which is likely important for the extensive homologue pairing that takes place in synaptonemal complex deficient meiocytes. In contrast, MSH4 foci counts remained low in *Mcmdc2*^*-/-*^ meiocytes, indicating an earlier impairment in recombination that provides a likely reason for the observed failure in homologue alignment. Thus, defective synaptonemal complex formation cannot account for the observed defect in recombination in *Mcmdc2*^*-/-*^ meiocytes. These observations strongly indicate that defective synaptonemal complex formation is the consequence of failed recombination in *Mcmdc2*^*-/-*^ meiocytes, and not vice versa. Consistent with this conclusion, we found that MCMDC2 was not required for synaptonemal complex formation in the DSB formation defective *Spo11*^*-/- *^spermatocytes, which form extensive non-homologous synapsis. This observation suggests that MCMDC2 is not involved directly in synaptonemal complex formation, although we cannot formally exclude the possibility that MCMDC2 plays a direct role specifically in homologous synaptonemal complex formation in a DSB formation-proficient background. Synaptonemal complex formation was much more limited in *Mcmdc2*^*-/-*^ than in the DSB formation defective *Spo11*^*-/-*^ and *Spo11*^*-/-*^ *Mcmdc2*^*-/-*^ meiocytes. Interestingly, synaptonemal complex formation is also reduced in strand-invasion defective *Dmc1*^*-/-*^ and *Hop2*^*-/-*^ meiocytes as compared to the DSB formation defective *Spo11-*deficient meiocytes [60]. Thus, accumulation of SPO11-dependent recombination intermediates may interfere with “erroneous” non-homologous synaptonemal complex formation in mutants where homologue pairing is defective due to an early block in recombination. Recombination intermediates might also inhibit non-homologous synaptonemal complex formation in unperturbed meiosis, which could help to ensure that synaptonemal complexes form between homologous substrates. Unrepaired DSBs may inhibit non-homologous synaptonemal complex formation directly. Alternatively, unrepaired DSBs may have an indirect effect by altering cell cycle-progression. Although, spermatocytes are eliminated in stage IV testis tubules in both DSB repair defective (e.g. *Dmc1*^*-/-*^, or *Mcmdc2*^*-/-*^) and the DSB formation defective *Spo11*^*-/-*^ spermatocytes, it has been proposed that *Spo11*^*-/-*^ spermatocytes progress further in meiotic prophase [55,73]. This is because the mid-late pachytene marker histone H1T was observed in *Spo11*^*-/-*^ spermatocytes but not in DSB repair defective spermatocytes, which indicates that unrepaired DSBs likely delay progression through meiotic prophase [55,73].

### What is the function of MCMDC2 in recombination?

The accumulation of RAD51 and DMC1 foci in *Mcmdc2*^*-/-*^ meiocytes indicates that DNA ends were resected, and single-stranded DNA overhangs recruited strand-invasion promoting recombinases at break sites. Yet, these inferred RAD51 and DMC1 coated single-stranded overhangs were unable to efficiently promote homologue pairing. This might indicate that RAD51 and DMC1 recombinases cannot promote strand invasions effectively in the absence of MCMDC2. Alternatively, strand invasions and D-loop formation may still occur, but these recombination intermediates are not stabilized sufficiently to ensure alignment of homologues and the formation of extensive synaptonemal complexes. The observation that MSH4 foci numbers are low in *Mcmdc2*^*-/-*^ meiocytes is consistent with both of these scenarios. In the former scenario, recombination intermediates that could recruit the MutSγ complex would not form in *Mcmdc2*^*-/-*^ meiocytes, hence MSH4 foci could not form either. In the latter scenario, accumulation of MutSγ complex at strand invasion intermediates/D-loops would be defective in *Mcmdc2*^*-/-*^ meiocytes. Hence, these recombination intermediates would be unstable and might be dissolved by helicases, e.g. the BLM helicase, which has been proposed to antagonize MutSγ in its function of stabilizing recombination intermediates [31,35]. It follows, that MCMDC2 would play an important role in MutSγ function in the latter scenario.

MCM proteins are AAA+ ATPases that form hexameric rings on duplex DNA and promote the melting of double-stranded DNA in an ATP dependent-manner (reviewed in [40]). MCM2-7 are primarily involved in the initiation of DNA replication [74], but MCM8 and MCM9 are particularly important for homologous recombination in mitotic cells in vertebrates [45–48]. MCM8 and MCM9 interact, and are thought to also form hexameric helicase complexes [45,46,48]. MCM8 and MCM9 promote DNA repair by facilitating resection of DNA ends at DSB sites [47], promoting an as yet undefined post-strand-invasion steps of recombination [45,46], and melting DNA at sites of mismatches during mismatch repair in somatic cells [75]. Despite being crucial for recombination and mismatch repair in mitotically dividing cells, MCM9 does not have an essential role in meiotic recombination [45,76]. Thus, MCM8 functions independent of MCM9 in meiosis. The *Drosophila* orthologues of MCM8 and MCMDC2 form a protein complex that is presumed to stabilize strand invasion intermediates of recombination specifically in meiosis [43]. Furthermore, the meiotic phenotypes of *Mcm8*^*-/-*^ [45] and *Mcmdc2*^*-/-*^ mice appear very similar, although no data was reported on MutSγ behavior in *Mcm8*^*-/-*^. Thus, it is tempting to speculate that MCM8 and MCMDC2 collaborate in mouse meiosis, and that MCMDC2 replaces MCM9 in MCM8-containing helicase complexes in meiosis. Curiously, the sequences of Walker A and B motifs, which are domains that are necessary for the ATPase activity of MCMs, are apparently not conserved in either *Drosophila* or mammalian MCMDC2 proteins [43]. This suggests that MCMDC2 is unlikely to function as an ATPase, but it may function as a modulator in putative meiotic helicase complexes that contain other MCMs (e.g. MCM8) with an active ATPase domain. Interestingly, the *Drosophila* MCM8 orthologue, REC, was proposed to facilitate repair DNA synthesis during meiotic recombination, because meiotic gene conversion tracks were significantly shortened in *rec* mutants [41]. Putative mammalian MCM8/MCMDC2-containing helicase complexes may have similar functions. One possibility could be that MCMDC2 promotes unwinding of the invaded DNA at sites of strand invasions. This could facilitate the formation of extended strand invasions, and/or may be needed for efficient DNA repair-synthesis starting from the 3´end of the invading strands. These hypothesized functions would be expected to stabilize strand invasions. Unwinding invaded homologous sequences to promote the formation of extended and stable D-loops might be particularly important for inter-homologue recombination during meiosis. The reason is that mismatches that can occur between homologues would likely interfere with extension of D-loops thereby antagonizing the stabilization of inter-homologue strand-invasion intermediates.

### MCMDC2 and MutSγ

The observation that MCMDC2 is required for the accumulation of MSH4 at recombination intermediates during meiosis raises the interesting possibility that a putative MCMDC2-containing helicase complex and the MutSγ complex may physically interact and collaborate in stabilizing D-loops. At sites of DNA mismatches, MCM9 forms a complex with MSH2 and MSH6, which are homologs of MutSγ components MSH4 and MSH5, and the complex of these proteins is thought to be crucial for correct mismatch repair in mitotically active cells [75]. Thus, it is possible that functional and/or physical interaction between MSH proteins and “DNA repair-promoting” MCMs is a conserved principle in distinct DNA repair pathways. Relevant to this point is the observation that the MutSγ complex is missing from *Schisophora*, a taxon that includes *Drosophila* [43]. It has been proposed that a complex of REC (*Drosophila melanogaster* MCM8) and MEI-217/218 (two *Drosophila melanogaster* orthologues of MCMDC2) proteins substitutes for the functions of the missing MutSγ complex in antagonizing BLM helicase, stabilizing strand invasion intermediates, and promoting crossover formation in meiosis in *Drosophila* [43]. The REC/MEI-217/218 complex may have been capable of replacing MutSγ in *Schisophora* because MCMDC2-containing complexes and MutSγ might have interacted and had shared functions in stabilizing DNA strand invasion intermediates in ancestral taxa where both of these complexes existed. Thus, loss of MutSγ in *Schisophora* may have required only a modification to an already pre-existing (and possibly conserved) function in MCMDC2-containing complexes. This speculative scenario would be certainly consistent with a putative conserved functional interplay of MutSγ and MCMDC2 in stabilizing recombination intermediates during mammalian meiosis. Thus, an important aim of future studies of MCMDC2 functions will be to address if MCMDC2 forms helicase complexes with other MCMs and if these complexes interact physically and functionally with MutSγ to stabilize D-loops.

## Materials and Methods

### RNA-isolation and RT-PCR

To test *Mcmdc2* expression in testes of wild-type and *Mcmdc2*^*-/-*^ mice, RNA was isolated and RT-PCR was performed as described earlier [20,77]. The RNA of the somatic tissue mix in (Fig 1a) originated from 17 distinct tissues: liver, brain, thymus, heart, lung, spleen, kidney, mammary gland, pancreas, placenta, salivary gland, skeletal muscle, skin, small intestine, spinal cord, tongue and uterus. The sequence of transcript-specific primers for RT-PCR were:

Mcmdc2 1R (Fig 1a) 5’-CGTTCCCTGTTGCAGTCTCT

Mcmdc2 1F (Fig 1a) 5’-CCCCACACAGCAAAAGTTCC

s9for (Fig 1a) 5’-GGCCAAATCTATTCACCATGC

s9rev (Fig 1a) 5’-TAATCCTCTTCCTCATCATCAC

Mcmdc2 Exon3 fw (Fig 1d, exon 3/4) 5’-ATTCAAAGCAGAGTTATGCTG

Mcmdc2 Exon4 rv (Fig 1d, exon 3/4) 5’-TTGAGTTTCAGTCTGTAACTGT

Mcmdc2 Exon5 fw (Fig 1d, exon 5/6) 5’-ATCAATATTGTGCTGAAGTTAAC

Mcmdc2 Exon6 rv (Fig 1d, exon 5/6) 5’-ACCAAGTACTCTAAATTTTCTGT

Mcmdc2 Exon6 fw (Fig 1d, exon 6/7) 5’-GATTTCAGTATGTGAGAGTCC

Mcmdc2 Exon7 rv (Fig 1d, exon 6/7) 5’-CTCTTAGGAAAATACCAAGTGA

Mcmdc2 Exon8 fw (Fig 1d, exon 8/9) 5’-ATGAACTAGTGAATAAGATGAAAA

Mcmdc2 Exon9 rv (Fig 1d, exon 8/9) 5’-CTGTCTACAAGCAGAGTGTC

Mcmdc2 Exon10 fw (Fig 1d, exon 10/11) 5’-ACTTTTGAATTTTAGCATGAATCT

Mcmdc2 Exon11 rv (Fig 1d, exon 10/11) 5’-CATCTGACCAATCAGAGTACT

### Generation of knockouts and genotyping

*Mcmdc2* was targeted in JM8A3.N1.C2 embryonic stem (ES) cells by the EUCOMM-IKMC project (project: 93238, ES line:HEPD0781_2_C06 and project: 118859, ES line: HEPD0800_2_F07). Targeting was based on a so called ‘knockout first’ multipurpose allele strategy [78] (Fig 1). Chimeras were generated by laser assisted C57BL/6 morula injections with ES cell clones heterozygote for the *Mcmdc2* ^insertion^ allele (Fig 1c). Progeny of the chimeric animals were crossed to the outbred wild-type CD-1 mouse line, and to pCAGGs-FLPo [79] and PGK-Cre [80] transgenic mice to generate *Mcmdc2* ^*restored*^, *Mcmdc2* ^*deletion*^, and *Mcmdc2* ^*insertion–deletion*^ alleles from the *Mcmdc2* ^*insertion*^ allele (Fig 1b). Mice were maintained on the outbred ICR (CD-1) background. Mice were genotyped by PCR using tail-tip genomic DNAs. Genotyping primers:

LacZfor 5’-TGGCTTTCGCTACCTGGAGAGAC

LacZrev 5’-AATCACCGCCGTAAGCCGACCAC

CreFw 5’-GCCTGCATTACCGGTCGATGCAACGA

CreRv 5’-GTGGCAGATGGCGCGGCAACACCATT

FlpOFw 5’-GCTATCGAATTCCACCATGGCTCCTAAGAAGAA

FlpORv 5’-CAATGCGATGAATTCTCAGATCCGCCTGTTGATGTA

o566 5’-GCAAGAAAACTATCCCGACC

o1046 5’-CACAGTGAGGCCCAATATAAA

o1047 5’-TCCACAGGAAAAGGCAAACG

o1049 5’-GGTGCTAGCCCCTTCCTTTT

o1050 5’-TCACTTGGTATTTTCCTAAGAG

o1147 5’-TGAAAGTTGATATGAAACTGTATA

o1163 5’-AAGGTTGTAGAATTACAGCAGC

PCR product sizes: with LacZfor/LacZrev primers, *Mcmdc2*^*insertion*^ and *Mcmdc2*^*insertion–deletion*^ allele 208 bp, other alleles-no specific product; with o1046/o1047 primers, wild-type allele 244 bp, *Mcmdc2*^*insertion*^ allele 225bp, *Mcmdc2*^*restored*^ allele 225 bp, *Mcmdc2*^*deletion*^ allele no product, *Mcmdc2*^*insertion–deletion*^ allele no product; with o566/o1047/o1050 primers, wild-type allele 561bp, *Mcmdc2*^*insertion*^ allele 542bp, *Mcmdc2*^*restored*^ allele 542bp, *Mcmdc2*^*deletion*^ allele no product, *Mcmdc2*^*insertion–deletion*^ allele 751bp; with o1049/o1147 primers, wild-type allele 310bp, *Mcmdc2*^*insertion*^ allele effectively not amplifiable product (7370 bp), *Mcmdc2*^*restored*^ allele 467bp, *Mcmdc2*^*deletion*^ allele no product, *Mcmdc2*^*insertion–deletion*^ allele no product; with o1049/o1163/o1147 primers, wild-type allele 310 bp (and 3421bp), *Mcmdc2*^*insertion*^ allele effectively no amplifiable products (7370bp and 10462bp), *Mcmdc2*^*restored*^ allele 467bp (and 3559bp), *Mcmdc2*^*deletion*^ allele 690 bp, *Mcmdc2*^*insertion–deletion*^ allele effectively not amplifiable product (5683bp). FlpOFw/FlpORv were used to detect FlpO recombinase transgene (1500 bp), CreFw/CreRv were used to detect Cre recombinase transgene (750 bp).

### Animal experiments

Mice carrying *Spo11-*null alleles were described earlier [6,7]. Histology in testis, analysis of RAD51/DMC1 foci or the synaptonemal complex were carried out in mice lines derived from both independent clones. The phenotypes of all the listed alleles were examined. No obvious differences were detected in testis histology, RAD51/DMC1 foci accumulation and synaptonemal complex formation between mice derived from the different ES clones and between the *Mcmdc2* ^*insertion/insertion*^, *Mcmdc2* ^*deletion/deletion*^, and *Mcmdc2* ^*insertion–deletion/insertion–deletion*^ strains. We chose the HEPD0800_2_F07 derived *Mcmdc2* ^*insertion–deletion*^ allele for complete phenotypic analysis, hence this line was used in all the reported experiments. Given that *Mcmdc2* ^*insertion–deletion/insertion–deletion*^ mice lack three exons, which causes a frameshift we refer to this genotype as *Mcmdc2* ^-/-^. *Mcmdc2* ^*restored*/ *restored*^ mice were fertile and their spermatocytes were indistinguishable from wild-type spermatocytes reconfirming the specificity of the observed phenotypes in the *Mcmdc2* ^*insertion/insertion*^, *Mcmdc2* ^*deletion/deletion*^, and *Mcmdc2* ^*insertion–deletion/insertion–deletion*^ strains. Whenever possible, experimental animals were compared with littermate controls or with age-matched non-littermate controls from the same colony. All animals were used and maintained in accordance with the German Animal Welfare legislation (“Tierschutzgesetz”), the Directive 2010/63/EU of the European Parliament and of the Council on the protection of animals used for scientific purposes and its German implementation (Tierschutz-Versuchstierverordnung–TierSchVersV). All procedures pertaining to animal experiments were approved by the Governmental IACUC ("Landesdirektion Sachsen”) and overseen by the animal ethics committee of the Technische Universität Dresden. The license numbers concerned by the present experiments are DD24-5131/287/1 and 24–9168.24-1/2006-13 (tissue collection without prior *in vivo* experimentation).

### Growth curve measurements

Wild-type and *Mcmdc2*^*-/-*^ mouse embryonic fibroblasts were derived from 12.5–14.5dpc embryos using standard procedures [81]. We plated 10,000 mouse embryonic fibroblasts in triplicates in 1ml DMEM (GIBCO) in 24-well plates. Live cells were counted using Miltenyi Biotec MACSQuant on day 3, 6, 9, 13 and 15 of cultures without aphidicolin. For aphidicolin treatment, 10000 cells were plated and incubated with media containing 1μM aphidicolin for the first 24 hour of the culture. Live cells were counted on day 5, 9, 13, 17 after plating. The media was changed every 3rd day for both types of cultures.

### Antibodies

In addition to antibodies that were previously described [20,21] we used two commercial antibodies: mouse anti-MLH1 (IF 1:50, BD Biosciences, order number 551092) and rabbit anti-MLH1 (IF 1:50, Calbiochem, order number D00122409). We also used a chicken anti-SYCP3 antibody that was raised against a His-tagged version of a 99 amino acid-long (from 13E to 111E amino acids) peptide of SYCP3, which we overexpressed in *Escherichia coli* and purified using metal ion affinity chromatography. IgYs from the yolk of eggs of immunized chicken were extracted using a published protocol [82]. Anti-SYCP3 IgYs were affinity purified on immunizing-antigen coupled NHS-Activated Sepharose 4 Fast Flow beads (Cat#17-0906-01, Amersham, GE Healthcare) according to standard methods [83].

### Immunofluorescence microscopy

Preparation and immunostaining of testis-ovary cryosections and nuclear surface spreads of meiocytes were carried out as described before [20,21,84,85]. Recombination foci and synaptonemal complex stretches were counted manually on matched exposure images with the use of the count tool of Photoshop CS5. We counted anti-RAD51, -DMC1, -MSH4 or -SYCP1 signals that were associated with SYCP3-marked chromosome axes, to avoid counting signals that do not represent genuine recombination foci (RAD51, DMC1 and MSH4) or synaptonemal complexes (SYCP1).

### Statistics

Statistical analysis was carried out with GraphPad Prism 5. For the comparison of independent samples, the two-tailed non-parametric Mann_Whitney (two-sample Wilcoxon rank-sum) test was used.
